# Controllable fabrication of PS/Ag core-shell-shaped nanostructures

**DOI:** 10.1186/1556-276X-7-580

**Published:** 2012-10-23

**Authors:** Chunjing Zhang, Xianfang Zhu, Haixia Li, Imran Khan, Muhammad Imran, Lianzhou Wang, Jianjun Bao, Xuan Cheng

**Affiliations:** 1 China-Australia Joint Laboratory for Functional Nanomaterials, Xiamen University, Xiamen, 361005, China; 2 College of Materials, Xiamen University, Xiamen, 361005, China; 3 Physics Department, Xiamen University, Xiamen, 361005, China; 4 ARC Centre of Excellence for Functional Nanomaterials, University of Queensland, St. Lucia, Brisbane, Queensland, 4072, Australia; 5 State Key Laboratory of Polymer Materials Science and Engineering, Sichuan University, Chengdu, 610065, China

**Keywords:** PS/Ag core-shell-shaped nanostructures, Surface modification, *In situ* reduction

## Abstract

In this paper, based on the previous steps, a facile *in situ* reduction method was developed to controllably prepare polystyrene/Ag (PS/Ag) core-shell-shaped nanostructures. The crucial procedure includes surface treatment of polystyrene core particles by cationic polyelectrolyte polyethyleneimine, *in situ* formation of Ag nanoparticles, and immobilization of the Ag nanoparticles onto the surface of the polystyrene colloids via functional group NH from the polyethyleneimine. The experimental parameters, such as the reaction temperature, the reaction time, and the silver precursors were optimized for improvement of dispersion and Ag coat coverage of the core-shell-shaped nanostructures. Ultimately, the optimum parameters were obtained through a series of experiments, and well-dispersed, uniformly coated PS/Ag core-shell-shaped nanostructures were successfully fabricated. The formation mechanism of the PS/Ag core-shell-shaped nanostructures was also explained.

## Background

Recently, considerable effort has been devoted to controllable fabrication of nanostructured materials with tunable functional properties. One example is the fabrication of core-shell-shaped nanostructures. Such nanocomposite structures have many applications in different technological fields like bio-sensor, chem-sensor, electronics, catalysis, drug delivery, diagnostics, antibacterial agent, etc. [1-7]. Especially, fabrication of noble metal-coated latex core-shell-shaped nanostructures (CSSNs) is currently an attractive area of investigation. The noble metal nanoshells grown on dielectric core particles are of great interest due to their tunable optical properties from ultraviolet to near-infrared regions of the electromagnetic spectrum [8-13]. In this sense, fabrication of Ag-coated CSSNs is particularly of significance [14].

The properties of the Ag-coated CSSNs are dependent on metal coverage. Thus, control of metal coverage is important to the application of this kind of core-shell-shaped nanostructure [15]. Until now, various approaches have been reported to fabricate the Ag-coated CSSNs, such as electroless plating [16,17], layer-by-layer self-assembly [18], sonochemical methods [19], and so on. These methods have been attempted to coat uniform metal on the colloid cores. However, some disadvantages exist during the synthesis. For example, in the electroless plating, metals or compounds such as gold, palladium, and SnCl_2_ are used to activate the core surface, but they remain as an impurity in the final product. The layer-by-layer self-assembly is too complicated and time-consuming. In the sonochemical method, the high-intensity ultrasound and removal of oxygen are necessary; otherwise, impurity like Ag_2_O can be observed.

In this paper, based on the previous investigations [20], an advanced, rapid, and low-cost *in situ* reduction method with optimized fabrication parameters was developed for the fabrication of polystyrene/Ag (PS/Ag) CSSNs. In the first step, monodispersed PS colloids as core particles were prepared by emulsion polymerization and then modified with polyethyleneimine (PEI) several times. Subsequently, Ag seeds were formed *in situ* and immobilized on the surface of PS colloids by adding AgNO_3_ or silver ammonia solution into the PEI-modified PS colloids. Finally, sodium citrate was added in the dispersion to increase thickness of the Ag shell. In this work, key experimental parameters were optimized to improve the dispersion and Ag coverage of PS/Ag CSSNs. Finally, well-dispersed and uniformly coated PS/Ag CSSNs were obtained. The thickness and coverage of the Ag shells can be easily controlled by changing the temperature, time, and silver precursors by which the properties of PS/Ag CSSNs can be tailored.

## Methods

### Reagents

Styrene, potassium pyrosulphate (K_2_S_2_O_3_), sodium citrate (C_6_H_5_O_7_Na_3_·2H_2_O), ethanol (C_5_H_6_OH), and sodium lauryl sulfate (CH_3_(CH_2_)SO_4_Na) were purchased from Shantou Dahao Fine Chemical Co., Ltd. (Shantou, Guangdong, China); silver nitrate (AgNO_3_), ammonia, PEI (MW600000-1000000) were purchased from Shanghai Chemicals Co. Ltd. (Shanghai, China). Water used during the experiments was distilled twice.

### Synthesis

**Figure 1:**
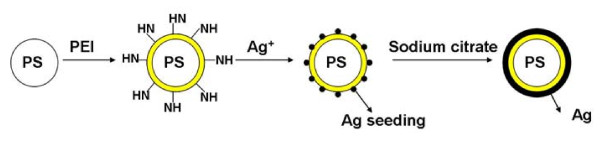
Schematic of PS/Ag CSSN synthesis procedure.

Figure 1 schematically presents the coating procedure. Following the synthesis of the PS colloids, functional group of NH was introduced to the surface of the PS colloids by PEI modification. The Ag ions were then added into the dispersion solution, and they become immobilized on the surface of the PEI-modified PS colloids. After stirring and heating the composite, the sodium citrate was added to increase the thickness of the Ag shell with the *in situ* reduction. Ultimately, the PS/Ag CSSNs were obtained. A detailed description is given as follows:

The monodispersed PS colloids for the coating cores were prepared by emulsion polymerization in aqueous alcohol system [21]. Potassium pyrosulphate (0.0490 g) and sodium lauryl sulfate (0.0538 g) were dissolved in 70-ml aqueous alcohol solution (volume ratio is 2:5) in a glass vial. After sealing in nitrogen gas, 2.2-ml styrene was added under nitrogen atmosphere and stirred rapidly. Then, the vial was submerged in a thermostatic oil bath and heated at 343 K for 8 h. The as-obtained PS particles with a diameter at about 450 nm were washed extensively with ethanol in centrifuge and dried in air at room temperature.

For the surface treatment, 0.5 ml of the PS colloids were dispersed into 30 ml of the deionized water containing 1 ml of 1% PEI under stirring and then the suspension was ultrasonically treated for 10 min. Following that, a further stirring was carried out for another 15 min to ensure the adsorption of PEI on the surface of the PS spheres. After that, the excess PEI was removed by centrifuging at 10,000 rpm for 10 min and then washed with water. The above procedure was repeated three times to obtain completely the PEI-modified PS colloids.

The silver ammonia solution was prepared by adding ammonia to silver nitrate solution until the precipitates dissolved completely. The chemical reaction is given below:

$${\text{AgNO}}_{3}+{2\text{NH}}_{3}\cdot {\text{H}}_{2}\text{O}\to \text{Ag}{\left({\text{NH}}_{3}\right)}_{2}\text{OH}+{{\text{NO}}_{3}}^{-}+\text{OH}$$

Finally, quantitative AgNO_3_ or silver ammonia solution was mixed with the dispersed solution containing PEI-modified PS colloids. The mixture was heated until the color changed from white to yellow. After cooling the mixture to certain temperature, sodium citrate was added and stirred under this temperature. The obtained composite was washed with the deionized water to remove excess PEI.

### Characterization

The size and morphology of the particles were characterized by field-emission scanning electron microscopy (FE-LEO-1530 SEM) operating at 20 kV. The chemical compositions of particles were examined by X-ray energy-dispersive spectra (EDS). Zetasizer Nano Essentials (Malvern Instruments, Westborough, MA, USA) was used to measure the size and potential of particles.

## Results and discussion

**Figure 2:**
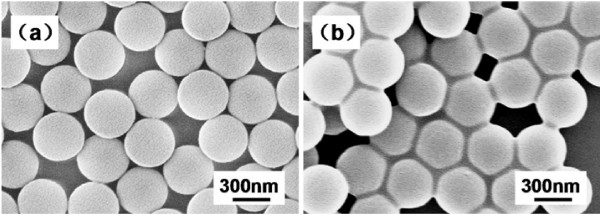
**SEM images.** PS core particles as fabricated (**a**) and after PEI surface modification (**b**).

Surface morphology of the original PS particles to serve as cores for the coating fabricated by emulsion polymerization is shown in Figure 2a. It can be seen that the obtained PS particles are spherical in shape with smooth surface. The average diameter and potential of PS particles as measured by ZS Nano Essentials were 450 nm and −34.5 mV, respectively. The core particles were monodispersed. The as-fabricated, monodispersed PS latex is essential for the fabrication of high-quality PS/Ag CSSNs.

Since these PS particles are covalently bonded whereas the nature of bonds in Ag is metallic, it is difficult to bond Ag atoms directly onto the PS surface. Therefore, surface modification with functional groups is essential for the purpose. Thus, cationic polyelectrolyte PEI was used to attach functional group of NH on the PS surface as shown in Figure 2b. The functional group of NH here mainly acts as a linker that easily coordinates with Ag ions in the solution. The polyelectrolyte PEI adsorption on the PS particles occurs spontaneously due to the driving force provided by electrostatic attraction, rendering a reverse of the surface charge of the PS particles. After the surface modification, the Zeta potential of the PEI-modified PS particles was measured to be 25.1 mV.

**Figure 3:**
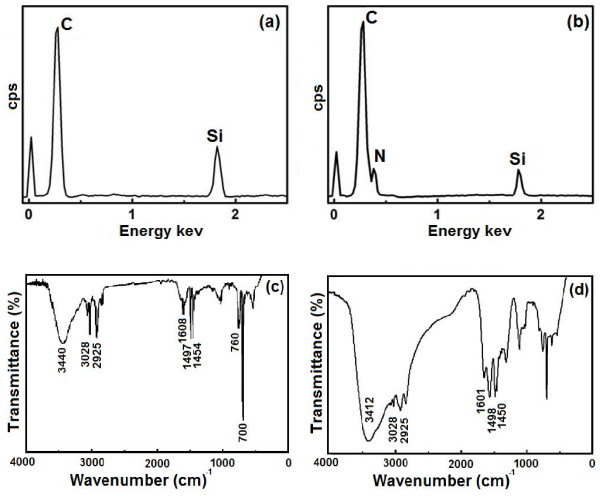
**EDS and FTIR spectra.** EDS spectra of PS particles (**a**) and PEI-modified PS particles (**b**) on Si substrate; FTIR spectra of PS particles (**c**) and PEI-modified PS particles (**d**).

Figure 3a,b shows the EDS analysis for chemical compositions of the PS particles and the PEI-modified PS particles. As shown in Figure 3a, except the peak of Si from the supporting substrate, there is only a strong absorption peak of carbon in the PS particles where the PS particles contain only carbon and hydrogen, and the hydrogen cannot be detected by EDS. Compared with Figure 3a, a weak peak for nitrogen appeared in the PEI-modified PS particles as given in Figure 3b, which illustrates the component of the functional group of NH in the PEI. Figure 3c,d gives a comparison between the infrared (IR) transmittance spectra of the PS particles and the PEI-modified PS particles. The absorption band at 3,440 cm^−1^ is due to the presence of hydroxyl group on the PS spheres, while the other absorption bands at 3,028, 2,925, 1,608, 1,497, 1,454, 760, and 700 cm^−1^ are due to the asymmetric, symmetric, and deformed vibrations of the -CH_2_ group of the benzene ring. It is obvious that the broad absorption peak at 3,412 cm^−1^ is typical of the N-H bond stretching vibrations, and peaks at 1,601, 1,498, and 1,450 cm^−1^ stem from stretching vibrations of the C-C bonds in the benzene ring [22]. The IR spectra indicate as well that the PEI has been attached on the PS particles.

**Figure 4:**
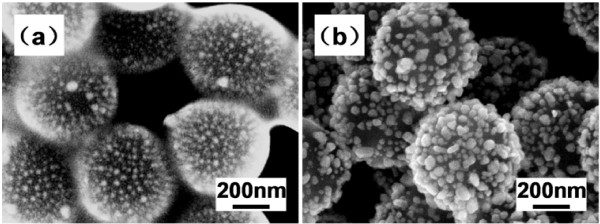
**SEM images of PS/Ag CSSNs at different stages of fabrication.** After seeding where Ag ions were coated on the surface of PEI-modified PS particles at 100°C (**a**); after reducing where Ag ions were reduced at 80°C (**b**).

**Figure 5:**
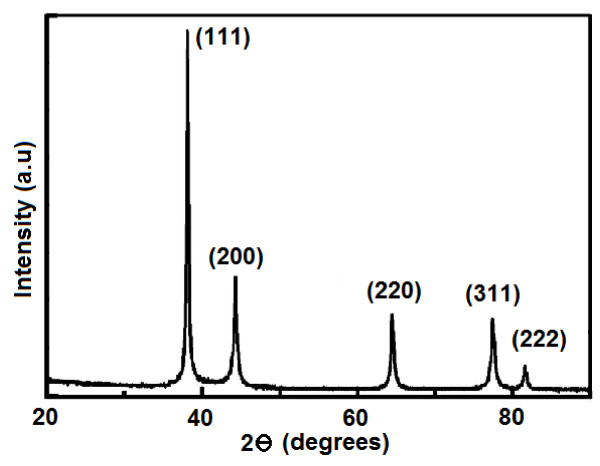
XRD patterns of PS/Ag CSSNs.

Figure 4 shows the SEM images of the PEI-modified PS particles coated with Ag at different stages. Figure 4a shows that during the seeding stage after the PEI modification, the Ag seeds uniformly coated on the surface of the PEI-modified PS colloids. However, the colloids adhered together due to the presence of the PEI (the case is the same for the adhesion of the PEI-modified PS colloids as shown in Figure 2b). It should be noted that although the PEI herein adsorbed on the PS core surface can bond with Ag ions via the immense interaction between the NH groups and the Ag ions, the PEI can also act as a reductant in the fabrication of Ag nanoparticles [20]. Figure 4b shows the final products of the PS/Ag core-shell-shaped nanostructures as obtained by reduction of the Ag^+^ ions with the sodium citrate. The products were washed by deionized water where the excess PEI was removed, and thus, the isolated core-shell structure can be obtained. We can see that the obtained PS/Ag CSSNs are well-dispersed, uniformly coated with a good coverage of Ag where the coated metallic Ag is not oxidized as confirmed in Figure 5.

**Figure 6:**
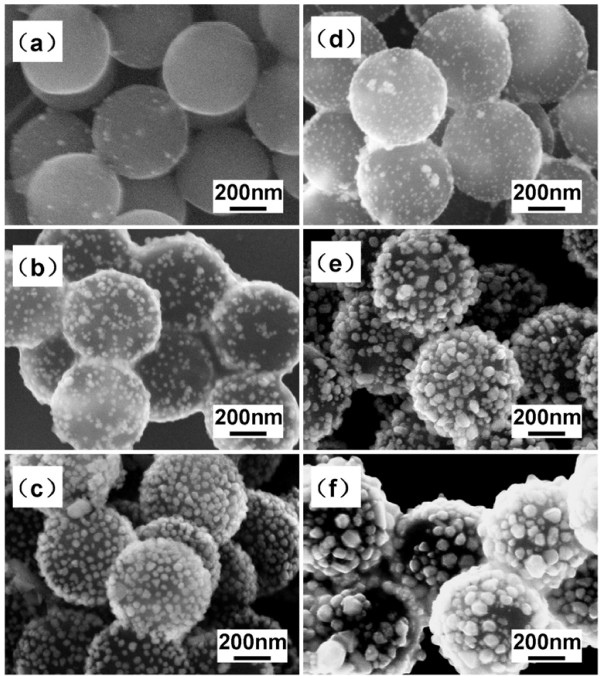
**SEM images of PS/Ag CSSNs fabricated at different temperatures of seeding and reduction.** (**a**) 60°C and 80°C, (**b**) 80°C and 80°C, (**c**) 100°C and 80°C, (**d**) 100°C and 70°C, (**e**) 100°C and 90°C, (**f**) 100°C and 100°C.

Our study shows that different fabrication parameters have obvious influence on the structure of the PS/Ag CSSNs. For this reason, we studied systematically crucial influences of the key parameters on the structure of the PS/Ag CSSNs. First, the influence of temperature was investigated, and the results are shown in Figure 6. In order to avoid any obvious glass transition of the PS, we studied the processing temperature not higher than 100°C. The AgNO_3_ was added into the PEI-modified PS colloids, stirred or seeded at different temperatures (60°C, 80°C, and 100°C) for 1 h, and subsequently reduced by sodium citrate for another 30 min at 80°C as shown in Figure 6a,b,c. We can see that the coverage of Ag nanoparticles was increased with the seeding temperature, which can be explained by crystal nucleation mechanism. It is well known that Ag was deposited on the surface of the PEI-modified PS cores by the reducibility of functional groups of NH. For this reason, Ag ions were reduced and formed crystal nuclei. However, only those nuclei which overcomed critical nucleation energy could become stable and able to grow. As we have known, the critical nucleation energy was decreased with the temperature. Therefore, the higher the temperature is, the more silver nanoparticles will be formed. Obviously, the seeding temperatures of 60°C and 80°C were too low for the seeding of the Ag nanoparticles onto the PS colloids, whereas the best seeding temperature is 100°C at which the PS cores were well coated.

Figure 6d,e,f shows the SEM images of PS/Ag CSSNs seeded with the same nucleation temperature at 100°C for 1 h but reduced or grew for 30 min with the sodium citrate at different temperatures (70°C, 90°C, and 100°C). We can see that the size of the Ag particles gradually increases with the reducing temperature. It indicates that the key factor affecting the crystal growth via reduction is the diffusion of ions in the solution. With the increase in temperature, the diffusion process becomes facile, and the size of Ag nanoparticles becomes larger. Nonetheless, the size of Ag nanoparticle is too large as reduced at temperature higher than 90°C which effects homogeneity of the PS/Ag CSSNs. As a consequence, the best coating process is seeding at 100°C and then reducing at 80°C.

**Figure 7:**
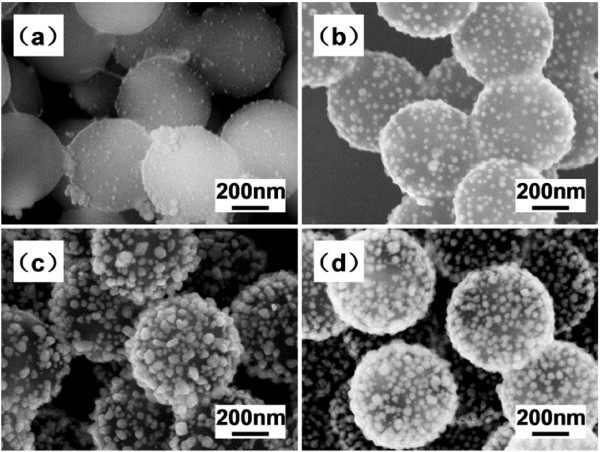
**SEM images of PS/Ag CSSNs.** Seeded at 100°C for (**a**) 20 min, (**b**) 40 min, (**c**) 1 h, and (**d**) 1.5 h.

The seeding time is another important factor in the fabrication, which was found to have a particular effect on the Ag coverage of the PS/Ag CSSNs. We can see this obviously from Figure 7. In Figure 7a, few Ag nanoparticles seeded, but they barely covered the PS cores in 20 min. As the time increased, more Ag nanoparticles nucleated, and the nucleated Ag nanoparticles further grew up. Thus, PS cores become more covered with the Ag nanoparticles as shown in Figure 7b,c. Finally, the change of the PS/Ag CSSNs' Ag coverage is not so obvious after 1 h later as seen in Figure 7d. The observation indicates that there were more Ag^+^ ions in the solution which could transport to the Ag crystal nuclei as the time increased. However, the system seemed to reach the state of saturation with the PEI after the modification in a certain time later. As a result, the best time for the Ag seeding for the advanced fabrication of the PS/Ag CSSNs should be longer than 1 h.

**Figure 8:**
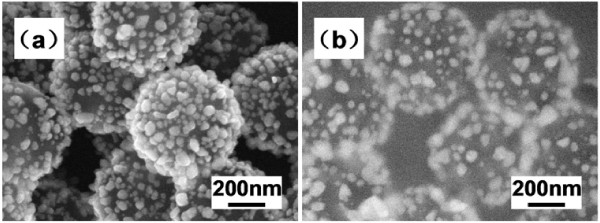
**SEM images of PS/Ag CSSNs.** Fabricated via seeding at 100°C for 1 h by different Ag ions: (**a**) AgNO_3_ and (**b**) silver ammonia solution.

The environment of the Ag^+^ in solution in many cases can be critically important for the rational control over surface and interfacial modifications of the molecular absorption process. The commonly used polyelectrolyte binders are very sensitive to their local ionic environment [23]. Our experiments demonstrated that when silver ammonia solution instead of silver nitrate was added into the PEI-modified PS colloids as shown by the comparison of Figure 8b,a, the core-shell-shaped structures easily adhered together and were not well coated. Therefore, Ag ions hydrolyzed from AgNO_3_ were used widely in the latest papers. The detailed mechanism for the difference is under a further exploration.

## Conclusions

The experimental results showed that the stirring of the PEI-modified PS colloids with AgNO_3_ at 100°C at least for 1 h followed by the reduction via sodium citrate at 80°C for 30 min leads to the well-dispersed, uniformly coated PS/Ag CSSNs. During the *in situ* reduction, the Ag seeds immobilized on the surface of the PEI-modified PS colloids through the linkage of functional group N-H. The dispersion and the Ag shell coverage of the PS/Ag CSSNs can be easily controlled by changing the temperatures of seeding and reduction, the seeding time, and the kind of silver ions. This optimized process is crucial to fabrication of PS/Ag CSSNs and their applications.

## Competing interests

The authors declare that they have no competing interests.

## Authors’ contributions

CZ performed the experiment, analyzed the data, and wrote the paper. XZ designed the experiment, analyzed the data, and wrote the paper. HL, IK, and MI helped write and revise the paper. LW and JB helped design the experiment. XC helped draft the manuscript. All authors read and approved the final manuscript.

## Authors’ information

CZ is an M.S. candidate in materials science and engineering in the China-Australia Joint Laboratory for Functional Nanomaterials at Xiamen University with her research interest focused on fabrication of nanomaterials. XZ is a Ph.D. in electronic materials engineering at the Australian National University and one of the earliest scientists who initialized nanoresearch in China. He is presently the director of the China-Australia Joint Laboratory for Functional Nanomaterials, an adjunct professor at The University of Queensland in Australia, and a full-time professor in the School of Physics and Mechanical and Electrical Engineering at Xiamen University in China, as well as the chief scientist for the AMAC International Inc., USA. He is the editor-in-chief of the *Scientific Journal of Physical Science*, associate editor of the *International Journal of Molecular Engineering* and is on the editorial board of several journals such as the *Chinese Science Bulletin*, etc. He is also an active referee for several top international journals such as *Applied Physics Letters*, *Journal of Physical Chemistry*, *Crystal Engineering Communications*, *Journal of Materials Chemistry*, and *Chinese Physics Letters*. His current research interests are focused on nanoinstabilities of low-dimensional nanostructures under external excitations, energetic beam nanoprocessing, controllable fabrication and growth of low dimensional nanostructures, controllable assembling and construction of large scale arrays of zero-dimensional nanostructures, organic and inorganic hybrid at nanoscale, functionalization of nanostructure, etc. He has authored and co-authored over 100 publications, filed 8 patents, chaired, co-chaired, or served as committee or advisory board member at over 20 international or national conferences, and presented over 60 invited lectures and talks at universities, research institutes, and major international conferences worldwide.HL is an engineer in the College of Materials at Xiamen University with her research interest focused on advanced energy materials.IK is a Ph.D. student in condensed matter physics in the China-Australia Joint Laboratory for Functional Nanomaterials at Xiamen University with his research interest focused on processing of nanomaterials. MI is a Ph.D. candidate in condensed matter physics in the China-Australia Joint Laboratory for Functional Nanomaterials at Xiamen University with his research interest focused on fabrication of nanomaterials. LW is a professor and research director in the ARC Centre of Excellence for Functional Nanomaterials at University of Queensland and a visiting professor in the China-Australia Joint Laboratory for Functional Nanomaterials at Xiamen University. His current research interest is concentrated on advanced functional nanomaterials. JB is an associate professor in the State Key Laboratory of Polymer Materials Science and Engineering at Sichuan University and a visiting associate professor in the China-Australia Joint Laboratory for Functional Nanomaterials at Xiamen University with his research interest in polymer materials. XC is a professor in the College of Materials at Xiamen University and a deputy director of the China-Australia Joint Laboratory for Functional Nanomaterials at Xiamen University with her research interest focused on advanced functional nanomaterials.
